# College Commencements and Alumni Association Meetings

**Published:** 1896-06

**Authors:** 


A Monthly Review of Medicine and Surgery.

*EDITORIAL STAFF, WOMEN'S EDITION:*

MAUD JOSEPHINE FRYE, M. D., *Managing Editor.*

ELECTA B. WHIPPLE, M. D. IDA C. BENDER, M. D.

HELENE KUHLMAN, M. D.

VOL. XXXV. JUNE, 1896. No. 11.

### COLLEGE COMMENCEMENTS AND ALUMNI ASSOCIATION MEETINGS.

### UNIVERSITY OF BUFFALO.

THE fiftieth commencement of the Medical Department of the University of Buffalo was celebrated in a becoming manner. Alumni hall greeted the assembled alumni on the morning of May 5th with a brave show of flags and blue and white bunting, the university colors. During the forenoon the usual business was transacted, the following officers being elected: president, P. W. Van Peyma, Buffalo; first vice-president, D. A. Currie, Englewood, N. J.; second vice-president, Herman G. Matzinger, Buffalo; third vice-president, J. A. McPherson, Tonawanda, N. Y.; fourth vice-president, J. W. Putnam, Buffalo; fifth vice-president, Charles Meine, Germania, Pa.; permanent secretary, E. L. Frost, Buffalo; recording secretary, N. V. Chappell, Buffalo; treasurer, H. U. Williams, Buffalo. One new trustee was elected, C. A. Wall, Buffalo. The executive committee remains as last year.

The committee on revision of the constitution, appointed last year, reported and after some discussion and amendment its report was adopted.

The afternoon session opened at 2 P. M. Dr. Baker, the outgoing president, being absent, Dr. Van Peyma occupied the chair.

The following scientific papers were read: Colpoperineorrhaphy, Henry J. Garrigues, M. D., New York; discussion by Drs. Eugene A. Smith and Charles H. Richmond. State of the gastric mucosa in secretory disorders of the stomach, Max Einhorn, M. D., New York; discussion by Drs. Charles G. Stockton, Allen Jones and A.

L. Benedict. Reconstructive surgery of the tubes and ovaries, Robert T. Morris, M. D., New York ; discussed by Drs. Henry J. Garrigues, M. D. Mann and C. C. Frederick. Some notes on the coronary arteries, George Dock, M. D., Ann Arbor, Mich.; discussed by Drs. H. R. Hopkins and DeLancey Rochester.

During the afternoon, Dr. M. D. Mann announced to the alumni the generous gift to the university by Dr. D. W. Harrington of $2,000 to establish the D. W. Harrington lectureship in the Medical Department. These lectures are to be given by men chosen by the medical faculty on such subjects as shall interest not only the advanced students in the university but the medical profession in general.

Dr. Charles G. Stockton laid before the alumni a plan concerning a suitable memorial to Dr. Thomas F. Rochester, which was explained in detail by Dr. H. U. Williams. It is proposed to raise a sum of money, the interest on which shall be used to maintain a fellowship in pathology, to be known as the Rochester memorial fellowship. This fellowship is to be awarded by competitive examination, to which the younger graduates of the university will be admitted.

GRADUATING EXERCISES.

The graduating exercises were held at Music hall at 8 P. M. After the opening prayer by Rev. William J. McKittrick, the candidates for the medical degree were presented to the chancellor by Dr. John Parmenter, secretary of the faculty. Their names are as follows: Byron A. Brown, M. D., Fairport, N. Y.; Marie L. Benoit, Montreal; Charles O. Boswell, A. B., Rochester; Normal L. Burnham, Buffalo; Clayton M. Brown, Weathersfield Springs; Albert H. Berry, B. S., Buffalo; George F. Craft, Gorham; William B. Cochrane, Penfield; Charles H. Cullinane, Watkins; Herman B. Cole, Hamburg; John Dugan, Geneva; James C. Dorr, Buffalo; Paul Dittman, Buffalo; Daniel A. Eiseline, Canandaigua; Charles V. Fairbanks, Harrisville; James W. Fox, Palmyra; R. Bruce Gamble, A. B., Meadville; Herman F. Graf, Buffalo; Addison P. Halsted, Potter; Charles D. Hauser, Girard; Mary M. Huntley, Buffalo; Horace L. Hulett, Little Genesee; Frank A Helwig, Clarence; James E. Holden, Buffalo; Elmer A. Jones, Warren, Pa.; John E. Jones, Buffalo; Regina Flood Keyes, Buffalo; William C. Keyes, Buffalo; W. L. Kistler, Buffalo; James E. King, Buffalo; Oscar H. Kraft, Buffalo; George A. Lane, Rochester; Charles E. Low, Pulaski; Guy L. McCutcheon, Ph. D.,

Buffalo ; William B. May, Buffalo ; Myron H. Metz, Williamsville ; Arthur A. Moore, Buffalo ; Charles G. Miller, Ph. D., Buffalo ; John M. Mesmer, Buffalo ; Charles E. Norris, Nunda ; Frederick C. Peterson, Watertown ; John K. Rupert, Buffalo ; William B. Reed, Ph. G., Buffalo ; Frederick E. Squires, East Avon ; Charles A. Shepard, Lewiston ; James W. Shaul, Jasper ; Harland L. Stanbro, Keuka College ; Joseph Spangenthal, M. D., Buffalo ; Francis I. Shepard, Buffalo ; Ulysses B. Stein, Buffalo ; James A. Taggart, Jr., Buffalo ; Alan P. Vaughn, Springville ; Arthur M. Whaley, Buffalo; Charles M. Whitcher, Ph. B., Mayville.

Following the administration of the Hippocratic oath by Dr. Mann, dean of the faculty, the chancellor, Hon. James O. Putnam, conferred the degrees.

Dr. Mann then announced the honor list of those who had an average of 90 and upward in medicine as follows : 1. Miss Mary M. Huntley ; 2. James E. King ; 3. Elmer A. Jones ; 4. Charles D. Hauser ; 5. Fred E. Squires ; 6. R. Bruce Gamble ; 7. Daniel A. Eiseline ; 8. Charles R. Cullinane ; 9. Charles G. Miller ; 10. Mrs. Regina Flood Keyes, Addison T. Halstead.

Honorable mention was accorded the thesis on The therapeutic effect of certain preparations of arsenic, by Guy L. McCutcheon. William C. Keyes received a check for $50 for the best thesis on Pathology, second prize going to Myron M. Metz.

The following students reached a general average, which makes them eligible to appointment as resident physicians at the Buffalo General Hospital : James E. King, Elmer A. Jones. Charles D. Hauser, Frederick E. Squires, R. Bruce Gamble, Daniel A. Eiseline, Charles R. Cullinane, Charles G. Miller, Addison T. Halstead, W. L. Kistler, William C. Keyes, Norman L. Burnam, Byron A. Brown, Frank A. Helwig, Clayton M. Brown, William B. Cochrane, Horace L. Hulett and Arthur M. Whaley. From the list the following were appointed : Dr. Charles G. Miller, Buffalo ; Dr. Charles D. Hauser, Girard, O. ; Dr. Norman L. Burnham, Buffalo; Dr. Byron A. Brown, Fairport, N. Y. If six appointments are made, Drs. King and Squires will also be made resident physicians at the hospital.

Dr. John R. Gray, secretary of the Department of Pharmacy, presented the class in pharmacy to Chancellor Putnam, who conferred the degree upon twenty-four students.

Dr. Willis G. Gregory, dean of the Pharmacy Department, announced that the William H. Peabody prize of $50 in the senior class had been won by John G. Brooks, of Ithaca. The honor roll of seniors is as follows: John G. Brooks, Charles E. Abbott, George F. Briggs, Ernest T. Sumner and Burt S. Stevens. Clifton C. Briggs, of Clifton Springs, was awarded the $25 prize in the junior class for laboratory and lecture work.

Dr. A. P. Southwick, secretary of the Dental Department, presented the candidates for the degree D. D. S., Dean Barrett administering the oath. The class numbered thirty-six.

The honor roll entitling its recipients to honorary places on the faculty was as follows: Henry D. Warren, Albert T. Lythe, Richard B. Redway, C. Arthur Bean and Henry F. Squire.

Chancellor Putnam then, in a brief but interesting manner, reviewed the history of the University from its organisation with Millard Fillmore as its first chancellor to the present. He said:

I well remember the interest and the hope that centered in the enterprise when a few of the citizens of Buffalo met to organise the university under its new charter. Its first chancellor was Millard Fillmore, who passed all the gradations of state and national honor. His last years were spent in his Buffalo home, representing every private and citizen virtue. His successor was O. H. Marshall, an able lawyer, a man of letters, who won distinction as an annalist of the early history of our West and Northwest. He was succeeded by the accomplished Sprague, who is gratefully remembered for his interest in and his labors on behalf of the university. The official relations of these three chancellors cover nearly the entire history of the university.

Following the organisation of the university its medical college was founded by a few of the leading physicians of the city. Three I may name were specially prominent in organising the college: Dr. James P. White, able, aggressive and courageous; Dr. Austin Flint, an unassuming, courtly gentleman, who, during his life in Buffalo and New York, attained great celebrity, largely contributing to the literature of his profession; and Dr. Frank H. Hamilton, a distinguished surgeon, ever gracious in manner, not carrying his points by storm, but winning his way by merit which could not be hid.

There were other strong members of the faculty, and the prestige given the college by its founders has never been lost. Their successors have not only maintained its early reputation, but have advanced the science of their specialties. Four other departments have been created during the last ten years: In January, 1886, the Department of Pharmacy, with 97 students now enrolled; in 1891, the Dental Department, already requiring a new building; in 1891, the addition of the Buffalo Law School; in 1895, the Department of Pedagogy. * * *

An academic department awaits the generous endowment which the wealth of Buffalo will undoubtedly in due time bestow. A great indus trial and commercial city has need of the inspirations which flow from these ideals that are in touch with the higher nature of man and of society.

Congratulations are in order, and I heartily extend mine to the official and educational departments of the university on the splendid record it has made in the first half century of its existence.

At the close Chancellor Putnam introduced the speaker of the evening, Mr. Joseph O'Connor, editor of the *Enquirer*, who delivered a most scholarly address which, did space permit, we should be glad to report in full. In closing he said: "There are some men who seem to be born physicians, and happy is he whom nature as well as the university faculty has given a commission to heal, in whom the impulse to serve is strong and sympathy for suffering intense. He has an instinct for the detection of disease like that of the setter for game. He has a soft touch and a strong will. He breathes confidence and challenges trust. He is cheerful but never blustering, and comes into a room like a wind from the pines in summer."

The dinner at the Genesee concluded the day. Dr. P. W. Van Peyma acted as toastmaster and the following toasts were responded to: The University of Buffalo, Dr. Roswell Park; Medical women, Dr. Ida C. Bender; The newspaper a sphere for the scientific man, Edward H. Butler; The patient, L. G. Sellstedt; The class of 1896, Dr. Charles G. Miller; The Buffalo General Hospital, Dr. Renwick R. Ross.

It is a noteworthy occurrence that at the fiftieth commencement of the Medical Department a woman should lead her class. Could those of half a century ago know this, what amazement would be theirs! And this is only the beginning. "What they have done, but the earnest of the things that they shall do."

---

NIAGARA UNIVERSITY.

THE eleventh annual meeting of the Alumni Association of the Medical Department of Niagara University was held at the college building, Tuesday, May 12, 1896. Dr. C. C. Frederick opened the meeting with an address of welcome, which was followed by the annual address of the president, delivered by Joseph J. Kane, M.D. The business session was then held and the officers for the ensuing year were elected as follows: President, Dr. J. S. Peterson, New York City; first vice-president, Dr. Frank W. Maloney, Rochester, N. Y.; second vice-president, Dr. J. W. Nash,

Buffalo ; secretary, Dr. Henry Osthues, Buffalo ; permanent secretary, Dr. John J. Twohey, Buffalo ; treasurer, Dr. Frederick M. Boyle, Buffalo ; executive committee, Dr. J. J. Twohey, Dr. F. A. Hayes and Dr. J. G. Ernest. The afternoon session was called to order at 3 P. M. by the president, Dr. Joseph J. Kane. Resolutions were read relative to the death of Dr. A. Victor Conley and Dr. Walter J. Riehl.

The first paper was read by Sidney A. Dunham, M. D., of Buffalo, who is making a special study of alcoholism; his subject was Involuntary intoxication from a medico-legal standpoint ; discussed by Drs. Harry A. Wood, L. J. McAdam and A. L. Benedict. The other papers were : Treatment of retrodeviations of the uterus, C. E. Congdon, M. D., Buffalo ; discussed by Dr. E. A. Smith ; Some heart lesions and their treatment, by D. L. Redmond, M. D., Buffalo ; discussed by Dr. A. L. Benedict ; Treatment of puerperal convulsions, with report of cases, L. G. Hanley, M. D., Buffalo ; and Puerperal eclampsia, with report of cases, by J. S. Peterson, M.D., New York. The last two papers were discussed by Drs. Ingraham, Lothrop, Buswell and Frederick.

The meeting was well attended and great interest evinced in the able papers presented. All of those reading papers were graduates of Niagara University.

GRADUATING EXERCISES.

The graduating exercises were held at the Star Theatre, at 8 P. M. On the stage were the members of the faculty, wearing their black gowns and scarlet hoods, and in the front rows sat twelve black-gowned graduates of the class of 1895-6, upon whom the degree of M. D. was about to be conferred. Their names are as follows : Joseph Patrick Francis Burke, Buffalo ; Cornelius John Carr, Greenwood ; Edward Charles Corston, Buffalo ; Frank Amos Crosby, Hickory Corners ; William Edward Goodsell, Medina ; Morgan Daniel Hughes, Elmira ; John Joseph Mahoney, Jamestown ; George Elias Nour, Syria ; James Albert Oliver, Stevensville, Ont.; Leib Hirshow Shenier, Buffalo ; Gideon Davis Smith, North Chemung ; James Stanislaus Walton, Scranton, Pa.

In opening the exercises Dr. Thomas Lothrop referred to the death of their beloved chancellor, the late Rt. Rev. Stephen Vincent Ryan, bishop of Buffalo. He announced that in the absence of the president of the University, the Rev. Father McHale, the trustees authorised Dr. John Cronyn, Ph. D., LL.D., president of the Medical Faculty, to confer the degrees.

The candidates, one at a time, were presented to the acting chancellor by Dr. H. C. Buswell, professor of the theory and practice of medicine, using the following Latin formula:

Insignissime Cancellarie: Presento tibi huncce scholarem in Facultate Medicinæ ut admittatur in gradum Doctoris in Medicina testorque eum quoad omnia quæ statuta requirunt aptum et idoneum esse.

The candidate then knelt before the seated chancellor, who took the hand of the graduate and pronounced the following words:

Ad profectum Republicæ ego, auctoritate mea:et totios Universitatis, admitto te ad gradum Doctoris in Medicina licentiamque tibi do omnia ea faciendi quæ ad illum gradum pertinent.

While this formula was reciting, Dr. Carlton C. Frederick, acting as beadle, "hooded" the graduate, who then rose and signed the Hippocratic Oath in the presence of Dr. A. A. Hubbell, secretary of the faculty.

The valedictory address was delivered by Herman Mynter, M. D., professor of operative and clinical surgery, and we would be glad to give it entire, did space allow. It was full of sensible advice; impressing upon the graduates the absolute necessity of keeping abreast of the times by close application and intelligent study; calling attention to the fact that medicine is a stern mistress and those who wish to succeed in that profession must bear in mind that advancement in the science of surgery and medicine is being made daily and success is achieved only by constant and well-directed efforts. It was listened to with rapt attention by the graduates and the large audience. After a graphic comparison of this profession with others, Dr. Mynter closed his address and gave way to Bishop Mallalieu.

In Bishop Mallalieu's eloquent address to the graduates he held before them the high ideals which should ever be the physician's.

The twelfth annual banquet was held at the Genesee. Covers were laid for about seventy. Dr. Joseph J. Kane acted as toastmaster. The toasts were as follows: Niagara University, Rev. L. A. Grace; The alumni, Dr. F. C. Gram; The beloved physician, Hon. R. B. Mahany; Our enemies, Mr. D. V. Murphy; The expert witness, Dr. W. C. Krauss; Stafford's motto, Mr. Joseph O'Connor; The gentlemen, Dr. Maud J. Frye; The ladies, Dr. H. C. Buswell; The newly-fledged, Dr. J. J. Mahoney.

This ended one of the most interesting commencement days in the history of Niagara University.

